# Loss of STAT3 in Lymphoma Relaxes NK Cell-Mediated Tumor Surveillance

**DOI:** 10.3390/cancers6010193

**Published:** 2014-01-27

**Authors:** Eva Maria Putz, Maria Agnes Hoelzl, Julia Baeck, Zsuzsanna Bago-Horvath, Christian Schuster, Brian Reichholf, Daniela Kern, Fritz Aberger, Veronika Sexl, Andrea Hoelbl-Kovacic

**Affiliations:** 1 Institute of Pharmacology and Toxicology, University of Veterinary Medicine, Veterinaerplatz 1, Vienna 1210, Austria; E-Mails: eva-maria.putz@vetmeduni.ac.at (E.M.P.); julia.baeck@gmx.at (J.B.); zsuzsanna.bago-horvath@vetmeduni.ac.at (Z.B.-H.); brian.reichholf@gmail.com (B.R.); veronika.sexl@vetmeduni.ac.at (V.S.); 2 Institute of Pharmacology, Center for Physiology and Pharmacology, Medical University of Vienna (MUV), Waehringer Strasse 13A, Vienna 1090, Austria; E-Mails: maria.holzl@ki.se (M.A.H.); christian.schuster@dkfz-heidelberg.de (C.S.); 3 Clinical Institute of Pathology, Medical University of Vienna (MUV), Waehringer Gürtel 18-20, Vienna 1090, Austria; 4 Department of Molecular Biology, University of Salzburg, Hellbrunnerstrasse 34, Salzburg 5020, Austria; E-Mails: daniela.kern@sbg.ac.at (D.K.); fritz.aberger@sbg.ac.at (F.A.)

**Keywords:** STAT3, lymphoma, BCR/ABL, NK cells, tumor immune surveillance

## Abstract

The transcription factors and proto-oncogenes STAT3 and STAT5 are highly activated in hematological malignancies and represent promising therapeutic targets. Whereas the importance of STAT5 as tumor promoter is beyond doubt, the role of STAT3 in hematological cancers is less well understood. Both, enforced as well as attenuated expression of STAT3 were reported in hematopoietic malignancies. Recent evidence implicates STAT3 as key player for tumor immune surveillance as it both mediates the production of and response to inflammatory cytokines. Here we investigated the effects of STAT3 deletion in a BCR/ABL-induced lymphoma model, which is tightly controlled by natural killer (NK) cells *in vivo*. Upon STAT3 deletion tumor growth is significantly enhanced when compared to STAT3-expressing controls. The increased tumor size upon loss of STAT3 was accompanied by reduced NK cell infiltration and decreased levels of the cytokine IFN-γ and the chemokine RANTES. Upon transplantation into NK cell-deficient mice differences in lymphoma size were abolished indicating that STAT3 expression in the tumor cells controls NK cell-dependent tumor surveillance. Our findings indicate that STAT3 inhibition in lymphoma patients will impair NK cell-mediated tumor surveillance, which needs to be taken into account when testing STAT3 inhibitors in preclinical or clinical trials.

## 1. Introduction

Tumorigenesis involves both tumor-intrinsic alterations as well as modulations of the tumor environment finally favoring tumor growth and maintenance. Transformation of a cell requires the expression of oncogenes or silencing of tumor suppressor genes [1]. Most malignancies remain dependent on the transforming oncogene, a process that is described as “oncogene addiction” and well described for oncoproteins such as BCR/ABL or EGFR [2]. Tumor cells may also acquire additional adaptations or changes in signaling pathways that they may become dependent on—a phenomenon called “non-oncogene addiction” [3]. Luo *et al*. hypothesized that interference with such signaling pathways would result in system failure and tumor cell death. Players of the JAK/STAT pathway—in particular STAT3 and STAT5—have been repeatedly shown to represent such critical factors [4,5,6,7,8].

STAT3 was shown to be persistently activated in a variety of solid and hematological cancers [5,6,9,10] and to promote metastasis [11,12,13]. Constitutive STAT3 activation relieves tumor cells from their dependence on cytokines and growth factors—thereby allowing continuous cell cycle progression and proliferation. Recently STAT3 was shown to support transformation via a metabolic function in mitochondria [14,15]. Concordantly, constitutively active STAT3 (STAT3αC)-expressing mice showed accelerated skin tumor formation upon UVB irradiation [16]. Moreover, crossing of MMTV-neu transgenic mice to STAT3αC-knock-in animals resulted in an earlier onset of mammary tumor formation accompanied by increased invasiveness [13]. Constitutive activation of the JAK/STAT pathway was described in patient-derived BCR/ABL^+^ leukemic cells [17,18,19,20]. The tumor promoting role of STAT3 in leukemia formation has been confirmed in mice: transduction of bone marrow (BM) with constitutively active versions of STAT3 rapidly induced leukemia in mice [21]. Tumor formation occurred spontaneously and in the absence of a driving oncogene: STAT3αC alone sufficed to induce leukemia. These observations prompted current investigations evaluating STAT3 as a prognostic biomarker in patients suffering from acute forms of leukemia (NCT01245231, NCT01138332, and NCT01057290). STAT3 inhibitors and antisense oligonucleotides are presently undergoing biosafety studies in clinical trials (NCT00955812, NCT00696176, NCT01563302, NCT01904123, NCT01423903, NCT01867073 and NCT01066663).

However, the role of STAT3 for tumor formation appears complex. A variety of studies described STAT3 as a tumor suppressor [21,22,23,24]. Ecker *et al*. observed a proliferative disadvantage of fibroblasts and suppression of myc-induced foci formation in the presence of STAT3αC [21]. In line, the subcutaneous injection of myc^+^STAT3αC^+^ fibroblasts resulted in a profound reduction of tumor weight. Moreover, Mair *et al*. described that conditional inactivation of STAT3 in hepatocytes and cholangiocytes aggravated bile acid-induced liver injury and fibrosis [25] thereby implicating a protective role of STAT3.

Tumor development is shaped and sculptured by external influences such as the surrounding immune system [26]. Immune cells continuously screen our body to detect and eradicate degenerated or transformed cells. Tumor surveillance is dominated by the interplay of myeloid cells, cytotoxic T lymphocytes (CTL) and natural killer (NK) cells. NK cells are the main players for the eradication of leukemic cells transformed by the BCR/ABL oncogene [27,28,29]. NK-dependent surveillance requires their recruitment, recognition and subsequent lysis of target cells. Attraction of NK cells is mediated by chemokines, such as CCL5 (or RANTES, binding CCR5), CCL19 and CCL21 (binding CCR7), CXCL10 (binding CXCR3) and CXCL12 (binding CXCR4) that are secreted by other immune cells or the tumor itself [30]. Recognition of tumor cells by NK cells is triggered by low MHC class I levels (“missing-self”) or enhanced expression of stress signals such as NKG2D ligands [31,32]. Receptor-receptor interactions between NK and target cells induce cytotoxicity that is either delivered through exocytosis of granules packed with lytic enzymes (e.g., perforin and granzymes) or by producing cytokines such as interferon (IFN)-γ stimulating adaptive immune responses.

STAT3 has many facets in cancer and may act as tumor promoter and tumor suppressor. STAT3 induces target gene transcription of pro-survival and proliferative genes such as *Bcl2* or *cyclin D1* that promote tumor growth and survival. Another layer of complexity comes from the fact that STAT3 is critically involved in the production of immune-modulatory cytokines such as IL-6, IL-10, IL-17 and IFN-γ [33,34] and pro-angiogenic factors like VEGF [35]. Persistent STAT3 activity may provoke synthesis of IL-10, IL-17 and VEGF. Moreover, STAT3 represents a critical player downstream of these cytokines. In summary, STAT3 is part of a feed-forward loop that results in immunosuppression and inflammation [36]. The inhibition or genetic ablation of STAT3 relieves immunosuppression and thus markedly ameliorates anti-tumor responses [36,37].

Given the delicate balance between STAT3’s tumor promoting and tumor suppressing actions it is critical to assess the net effect of STAT3 inhibition within the tumor and the impact on tumor surveillance. In this study we used a conditional *Stat3* knockout mouse model to investigate the consequences of *Stat3* deletion for BCR/ABL^p185+^-driven tumor growth. Intriguingly, we found that STAT3-deficient tumors were substantially larger, which was accompanied by reduced NK cell recruitment to the tumor. NK cell cytotoxicity is significantly lower against STAT3-deficient compared to STAT3-expressing tumor cells. In line with this, pro-inflammatory cytokines such as IL-6, IFN-γ, TNF-α and RANTES are markedly reduced in STAT3-deficient tumors.

## 2. Results and Discussion

### 2.1. Generation of STAT3-Deficient BCR/ABL^+^ Pro-B Cell Lines

**Figure 1:**
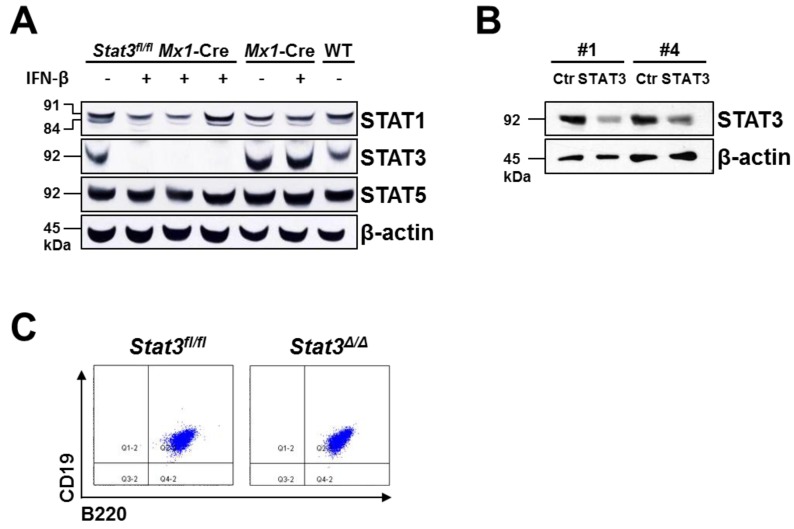
Generation of STAT3-deficient BCR/ABL^+^ pro-B cell lines. (**A**) Femur- and tibia-derived BM cells of *Stat3^fl^*^/*fl*^ *Mx1*-Cre and control mice were retrovirally infected with the BCR/ABL^p185^ oncogene. After the outgrowth of stable cell lines, *Stat3*-deletion was induced by the addition of 1,000 U/mL IFN-β and verified by western blotting. Loss of STAT3 was not compensated by STAT1 or STAT5 upregulation. β-actin served as loading control; (**B**) STAT3 was knocked down lentivirally in a v-ABL^p160+^ cell line (#1) and a BCR/ABL^p185+^ cell line (#4). Reduction of STAT3 was determined by western blotting; β-actin served as loading control; (**C**) Stable *Stat3^fl^*^/*fl*^* Mx1*-Cre (indicated as *Stat3^fl^*^/*fl*^) and *Stat3^∆^*^/*∆*^ cell lines expressed the typical pro-B cell markers CD19 and B220. One representative cell line is depicted.

Single cell suspensions of BM derived from *Stat3^fl^*^/*fl*^ *Mx1*-Cre and wild-type mice were infected retrovirally with the BCR/ABL^p185^ oncogene. After three weeks, stable growth-factor-independent BCR/ABL^p185+^ cell lines were established. Two independent approaches to delete STAT3 in these BCR/ABL^p185+^ cells were used. First, we deleted the endogenous *Stat3* locus in BCR/ABL^p185+^ *Stat3^fl^*^/*fl*^*Mx1*-Cre cell lines by IFN-β treatment (further on named *Stat3^∆^*^/*∆*^). Ten days thereafter loss of STAT3 was confirmed by western blotting. We also tested whether any other closely related STAT family members would compensate for the absence of STAT3. As depicted in Figure 1A protein levels of STAT1 and STAT5 were unaltered upon STAT3 deletion. In an additional approach STAT3 was reduced using lentiviral knockdown in two independently derived wild-type pro-B cell lines (#1 being transformed by v-ABL^p160^, #4 by BCR/ABL^p185^). This resulted in a STAT3 knockdown of 64% and 48% compared to control cells, respectively (Figure 1B). FACS analysis verified that the expression of B cell-specific markers (CD19 and B220) was unaltered upon loss of STAT3 (Figure 1C).

### 2.2. *In Vitro* Proliferation of BCR/ABL^+^ Cells Expressing or Lacking STAT3

**Figure 2:**
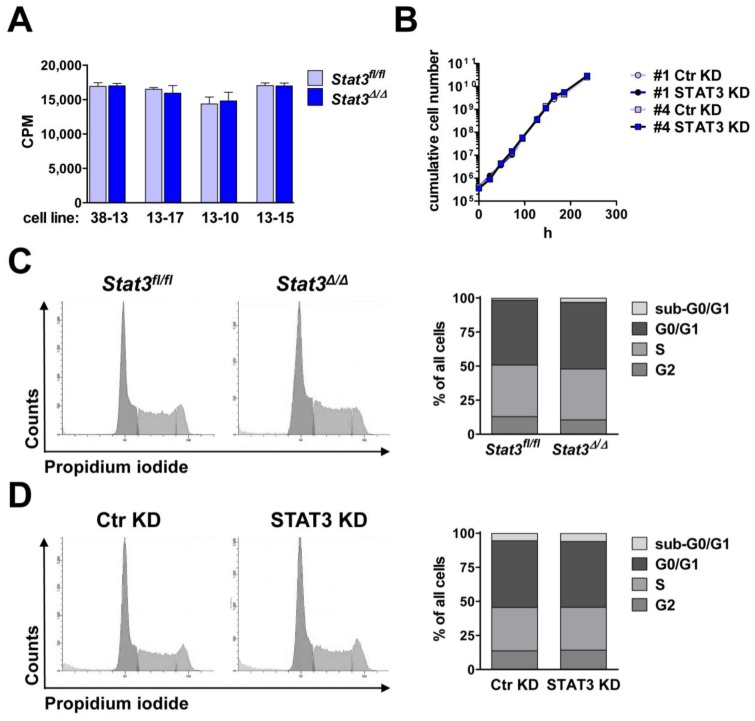
Comparable proliferation of BCR/ABL^+^ cells expressing or lacking STAT3. (**A**) [^3^H]-thymidine incorporation in stable *Stat3^fl^*^/*fl*^ and *Stat3^∆^*^/*∆*^ cell lines was identical (CPM = counts per min); (**B**) The growth curve of two different cell lines lentivirally infected with a hairpin targeting STAT3 (STAT3 KD) or a control hairpin (Ctr KD) were superimposable; A propidium iodide staining was performed and revealed identical cell cycle profiles of (**C**) a *Stat3^fl^*^/*fl*^ and corresponding *Stat3^∆^*^/*∆*^ cell line and (**D**) a cell line after STAT3 and control knockdown.

STAT3 is an important mediator of cell growth and survival [33,38,39]. Thus, inhibition of STAT3 in tumor patients has been proposed as a promising therapeutic strategy [40]. Together with STAT3, STAT5 was repeatedly described to be constitutively activated and to mediate tumor cell survival and proliferation [41,42,43,44]. As STAT3 and STAT5 share common anti-apoptotic and cell cycle-regulating target genes, it was speculated that they share redundant functions. However, in BCR/ABL-transformed cells survival depends on STAT5 but not on STAT3: deletion of *Stat5* resulted in a cell cycle arrest in G1 followed by apoptosis [8]. Apoptosis was not induced by deletion of *Stat3* [8]. We found that *Stat3^fl^*^/*fl*^ and *Stat3^∆^*^/*∆*^ cell lines showed superimposable proliferation rates (Figure 2A). Further, we could not detect any differences in proliferation between cell lines harboring a STAT3 KD or a control hairpin (Figure 2B). Accordingly, cell cycle profiles of STAT3-expressing and STAT3-deficient cells under normal culturing conditions were unaltered (Figure 2C,D).

### 2.3. Lymphoma Development *in Vivo*

**Figure 3:**
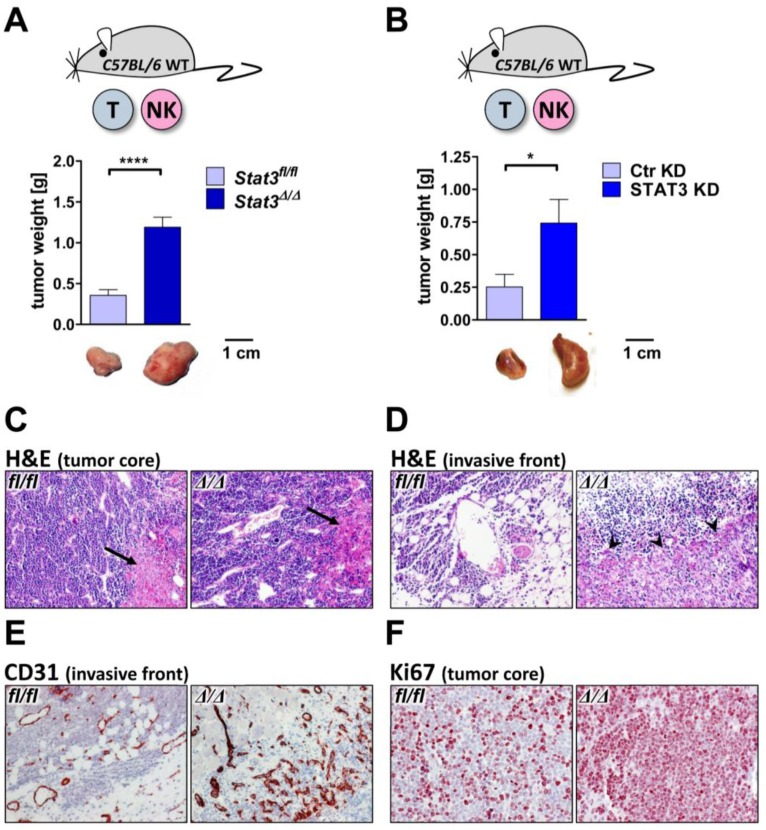
Loss of STAT3 in BCR/ABL^+^ lymphoma cells facilitates tumor growth. (**A**) 10^5^ *Stat3^fl^*^/*fl*^ or *Stat3^∆^*^/^*^∆^* BCR/ABL^p185+^ cell were injected subcutaneously into C57BL/6 mice. After 11 days tumors were dissected. STAT3-deficient lymphomas were significantly bigger compared to control lymphomas (*n* ≥ 6, *p* < 0.0001, unpaired *t*-test); (**B**) 10^5^ v-ABL^p160+^ cell expressing a hairpin targeting STAT3 (STAT3 KD) or a control hairpin (Ctr KD) were injected subcutaneously into C57BL/6 mice. Lymphomas derived from cells with STAT3 knockdown were significantly bigger than controls (*n* = 10, *p* = 0.029, unpaired *t*-test); (**C**) H&E stainings (10 ×) revealed a similar tumor architecture and necrosis (see arrows) within the tumor cores of *Stat3^fl^*^/*fl*^ or *Stat3^∆^*^/^*^∆^* lymphomas; (**D**) The tumor front showed a highly infiltrative behavior of *Stat3^∆^*^/^*^∆^* tumors and the occurrence of massive granulation tissue (see arrows), as determined by H&E staining (10 ×); (**E**) The edge of *Stat3^∆^*^/^*^∆^* tumors showed an accumulation of newly developed CD31^+^ blood vessels as expected from granulation tissue (10 ×); (**F**) Ki67 stainings (20 ×) unraveled clearly more proliferating cells in *Stat3^∆^*^/^*^∆^* tumors compared to *Stat3^fl^*^/*fl*^ controls. Asterisks denote statistical significance: * *p* ≤ 0.05; **** *p* ≤ 0.0001.

*In vitro* proliferation of transformed cell lines only partially reflects *in vivo* tumor formation. Within an organism tumor cells are exposed to a variety of cytokines that may affect survival and proliferation. Importantly the majority of these cytokines signals via the JAK/STAT cascade. STAT3 mediates target gene expression downstream of pro-inflammatory cytokines such as IL-6, TNF-α or IL-10 [45]. We thus speculated that despite the unaltered tumor cell proliferation *in vitro*, there may be a difference in tumor growth *in vivo*. To test this, *Stat3^fl^*^/*fl*^ and *Stat3^∆^*^/*∆*^ BCR/ABL^p185+^ cells were injected subcutaneously into the flanks of C57BL/6 wild-type mice. Intriguingly, after 11 days STAT3-deficient lymphoma sizes were significantly increased compared to wild-type tumors. The increased tumor size was evident irrespective whether the STAT3 abrogation was achieved genetically (Figure 3A) or by lentiviral knockdown (Figure 3B).

Staining of tumor sections with H&E, Ki67 and CD31 revealed obvious differences. Whereas we failed to detect any alterations in the degree of necrosis (Figure 3C), *Stat3^∆^*^/^*^∆^* tumor boundaries were less definite and the infiltrative character was more pronounced compared to *Stat3^fl^*^/*fl*^ controls (Figure 3D). The invasive front of *Stat3^∆^*^/^*^∆^* tumors was demarcated by formation of granulation tissue (Figure 3D, see arrows), which was also evident from the increased formation of nascent blood vessels at the marginal tumor area (Figure 3E). In contrast, STAT3-expressing tumors hardly harbored granulation tissue (Figure 3D,E). Within the tumor lymph and blood vessel densities were unaltered (data not shown). Ki67 staining unraveled a substantially enhanced proliferation of STAT3-deficient tumor cells *in vivo* (Figure 3F), which stands in clear contrast to our observations *in vitro*.

Taken together, deletion of STAT3 in BCR/ABL^+^ lymphoma resulted in an aggravation of tumor burden. At a first glance these results may be difficult to reconcile as they oppose studies by others, who defined STAT3 as a proto-oncogene [46,47]. Indeed there is growing evidence that STAT3 acts as a tumor suppressor in certain tissues [21,22,23,24]. Depending on the cellular system and on the oncogene that drives transformation STAT3 may thus be either considered a tumor suppressor or a tumor promoter. In the case of BCR/ABL^+^ lymphoma STAT3 acts as a tumor suppressor.

### 2.4. Cytokine Profile in STAT3-Deficient Lymphomas

The discrepancies between our *in vitro* and *in vivo* findings might result from the cytokine milieu that influences tumor growth *in vivo*. The involvement of STAT3 in cytokine signaling and production may contribute to the altered granulation tissue and blood vessel formation as well as cell proliferation in STAT3-deficient tumors.

**Figure 4:**
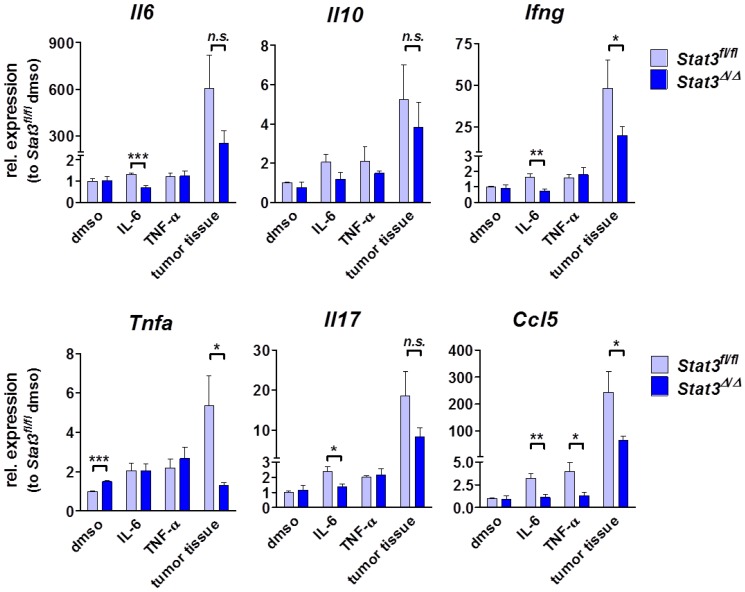
Cytokine expression profile *in vitro* and *in vivo*. mRNA levels were measured in a relative quantitative real-time PCR. For the *in vitro* measurements, three different cell lines (and their respective knockout counterparts) were used, either in control medium supplemented with dmso or after stimulation with 50 ng/mL IL-6 or 25 ng/mL TNF-α for 4 h. To determine the cytokine milieu within the lymphoma *in vivo*, RNA was prepared from tumors depicted in Figure 3A (*n* = 4–5 per group), meaning after transplantation of *Stat3^fl^*^/*fl*^ or *Stat3^∆^*^/*∆*^ cell lines subcutaneously into C57BL/6 wild-type recipients. Numbers indicate arbitrary units, relative to the expression in *Stat3^fl^*^/*fl*^ BCR/ABL^+^ cells cultivated under control conditions (dmso). Bar graphs depict means ± SEM (unpaired *t*-test). Asterisks denote statistical significance: * *p* ≤ 0.05; ** *p* ≤ 0.01; *** *p* ≤ 0.001; n.s. not significant.

We thus compared the expression levels of a number of candidate cytokines of *in vitro* growing and *ex vivo* derived tumor cells by real-time PCR concentrating on cytokines that either depend on STAT3 for production or signaling. The expression levels of IL-10, CXCL1, CXCL2, CCL2, CCL7 and VEGF were unaltered (Figure 4 and data not shown). In contrast, *Stat3^∆^*^/*∆*^ tumor tissue expressed reduced mRNA levels of IL-6 (607 *vs*. 256, *p* = 0.09), IL-17 (15.6 *vs*. 8.3, *p* = 0.09), IFN-γ (63 *vs*. 20, *p* = 0.012) and TNF-α (5.3 *vs*. 1.3, *p* = 0.031) (Figure 4). Previous reports have implicated the involvement of IFN-γ and TNF-α in proliferation and wound healing. Upon injury, IFN-γ- [48] as well as TNF-α-deficient animals [49] develop augmented granulation tissue and vessel formation. Accordingly, the reduced levels of IFN-γ and TNF-α in STAT3-deficient lymphomas are likely to contribute to the occurrence of granulation tissue and enhanced proliferation in these tumors.

Further, we found significantly lower expression of CCL5 (RANTES) in STAT3-deficient tumor cell lines *in vitro* as well as in STAT3-deficient lymphoma samples *ex vivo* (Figure 4). The chemokine CCL5 recruits and stimulates leukocytes to elicit anti-tumor immunity [50,51,52]. Further, CCL5 was described as a downstream target of TNF-α, which itself acts as a chemo-attractant for myeloid cells [53]. These findings prompted us to investigate the composition and amount of lymphoma-infiltrating leukocytes.

### 2.5. Lymphoma-Infiltrating Leukocytes

**Figure 5:**
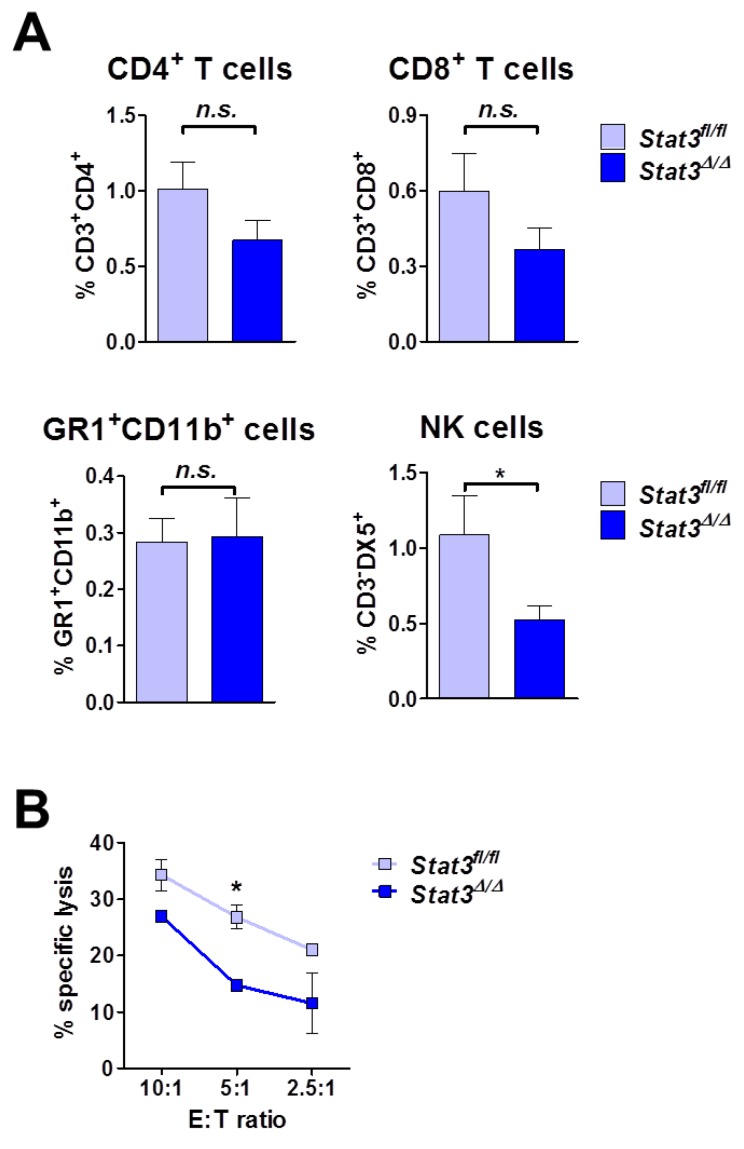
Immune cell infiltration and function in lymphoma. (**A**) *Stat3^fl^*^/*fl*^ and *Stat3^∆^*^/*∆*^ BCR/ABL^p185+^ cells were transplanted into C57BL/6 wild-type recipients. After 11 days single-cell suspensions of tumors were analyzed by flow cytometry for the presence of CD3^+^CD4^+^ and CD3^+^CD8^+^ T cells, as well as of GR1^+^CD11b^+^ myeloid and CD3^−^DX5^+^ NK cells. Bar graphs depict means ± SEM (*n* = 14; unpaired *t*-test); (**B**) NK cells were isolated from C57BL/6 wild-type splenocytes and co-incubated in different ratios with CFSE-labeled *Stat3^fl^*^/*fl*^ and *Stat3^∆^*^/*∆*^ BCR/ABL^p185+^ tumor cells, respectively. After 24 h, tumor cell lysis was determined by the addition of 7-AAD and flow cytometric analysis. Symbols represent means ± SEM, *n* = 2. Asterisks denote statistical significance: * *p* ≤ 0.05; n.s. not significant.

The majority of the cytokines we tested is produced rather by the tumor microenvironment than by the tumor cells themselves (Figure 4). This suggested that different numbers or an altered composition of lymphoma-infiltrating leukocytes may be present. As depicted in Figure 5A we indeed detected slight changes in the recruitment of CD4^+^ and CD8^+^ T cells in STAT3-deficient tumors that however did not meet the criterion of being statistically significant. Numbers of GR1^+^CD11b^+^ myeloid cells were comparable. Intriguingly, we found a statistically significant reduction in NK cells in STAT3-deficient tumors (1.1% ± 0.26% in *Stat3^fl^*^/*fl*^ *vs*. 0.52% ± 0.09% in *Stat3^∆^*^/*∆*^ tumors, numbers indicate means ± SEM, *n* = 14). As NK cells produce IFN-γ and TNF-α we reasoned that the decline of NK cell numbers in *Stat3^∆^*^/*∆*^ tumors accounts for the reduced levels of these cytokines (see Figure 4).

NK cells kill tumor cells without prior sensitization, which allows testing their cytotoxic ability *in vitro*. Indeed, STAT3-deficient tumor cells were killed less efficiently than their wild-type controls (Figure 5B).

These findings indicate that STAT3 expression in lymphoma cells is required to recruit NK cells as well as to evoke their cytotoxic capacity. NK cell cytotoxicity is regulated by a tight balance of inhibitory and activating signals transmitted from a plethora of different NK cell surface receptors [54,55,56]. When determining the levels of activating NKG2D—(MULT-1 and RAE-1) and NKp46-ligands as well as of inhibitory MHC class I (H-2K^b^ and H-2D^b^) on the lymphoma cells we failed to detect any differences in either of these ligands (data not shown). At present we lack definitive knowledge on the underlying mechanism that accounts for impaired NK cell cytotoxicity. We hypothesize that the reduced CCL5 levels in *Stat3^∆^*^/*∆*^ tumors contribute to this phenomenon as previous studies have described CCL5 as a NK cell activating chemokine [52]. Taken together, these data indicate that the enhanced lymphoma development after STAT3-deletion is associated with reduced NK cell infiltration and cytotoxicity.

### 2.6. Lymphoma Development in Immune-Compromised Recipients

As our findings show that *Stat3^∆^*^/*∆*^ tumors recruit and activate NK effector cells less efficiently, we tested lymphoma formation in mice lacking specific lymphocyte subsets.

**Figure 6:**
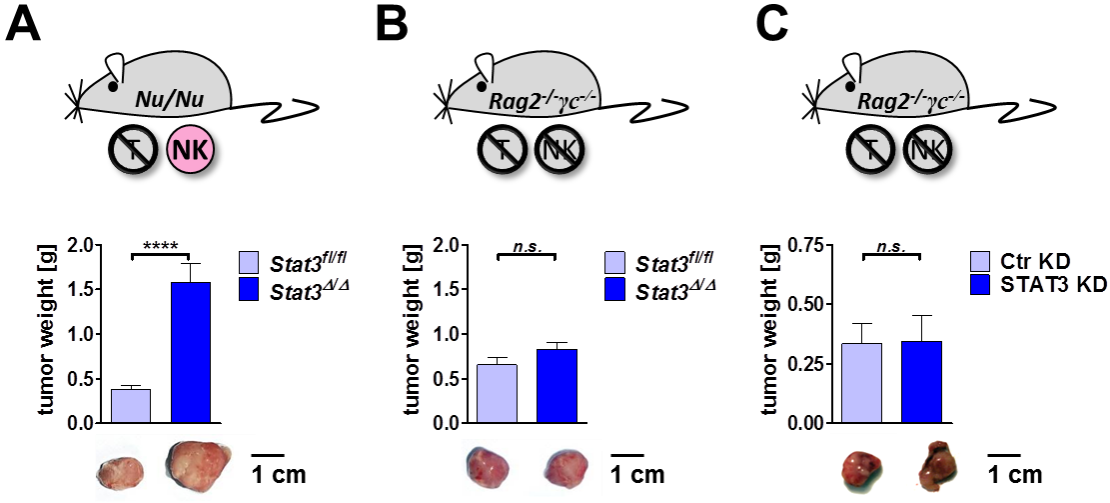
STAT3-dependent differences in tumor growth are lost upon transplantation in immune-deficient mice. (**A**) 10^5^ *Stat3^fl^*^/*fl*^ or *Stat3^∆^*^/*∆*^ BCR/ABL^p185+^ cells were injected subcutaneously into T cell-deficient *Nu*/*Nu* mice. After 11 days tumors were excised and weighted. STAT3-deficient lymphomas were significantly bigger than control lymphomas. Bar graphs depict means ± SEM (*n* ≥ 24, *p* < 0.0001, unpaired *t*-test); (**B**) 10^5^ *Stat3^fl^*^/*fl*^ or *Stat3^∆^*^/*∆*^ BCR/ABL^p185+^ cells were injected subcutaneously into *Rag2^−^*^/*−*^*γc^−^*^/*−*^ mice. 11 days after transplantation there was no difference in tumor weight. Bar graphs depict means±SEM (*n* = 18, *p* = 0.15, unpaired *t*-test); (**C**) 10^5^ v-ABL^p160+^ cell expressing a hairpin targeting STAT3 (STAT3 KD) or a control hairpin (Ctr KD) were injected subcutaneously into *Rag2^−^*^/*−*^*γc^−^*^/*−*^ mice. 11 days after injection, tumors developed equally well from both cell lines. Bar graphs depict means ± SEM (*n* = 10, *p* = 0.94, unpaired *t*-test). Asterisks denote statistical significance: **** *p* ≤ 0.0001; n.s. not significant.

We first transplanted *Stat3^fl^*^/*fl*^ and *Stat3^∆^*^/*∆*^ BCR/ABL^p185+^ tumor cells subcutaneously into *Nu*/*Nu* mice lacking T cells, but retaining functional B and NK cells for tumor immune surveillance. Comparable to the outcome of our studies in immune-competent wild-type mice (Figure 3A), transplantation of STAT3-deficient cells into *Nu*/*Nu* mice led to significantly enhanced tumor growth (Figure 6A). In contrast, upon transplantation into *Rag2^−^*^/*−*^*γc^−^*^/*−*^ mice that lack B, T and NK cells, the difference in tumor size was abrogated (Figure 6B). Comparable results were obtained upon transplantation of v-ABL^p160+^ cells expressing a hairpin targeting STAT3 (STAT3 KD) or a control hairpin (Ctr KD): No difference in tumor size could be detected after transplantation in *Rag2^−^*^/*−*^*γc^−^*^/*−*^ mice (Figure 6C). It is important to mention that the presence of NK cells is not the only difference in *Nu*/*Nu* and *Rag2^−^*^/*−*^*γc^−^*^/*−*^ mice. In contrast to *Rag2^−^*^/*−*^*γc^−^*^/*−*^ mice, *Nu*/*Nu* mice still harbor B cells. Additionally, *Rag2^−^*^/*−*^*γc*^−/*−*^ may differ in their tumor microenvironment due to the lack of cytokine signaling pathways that rely on the presence of the common γ chain such as IL-2, IL-4, IL-7, IL-9, IL-15 and IL-21 [57]. We cannot rule out that the altered cytokine milieu in *Rag2^−^*^/*−*^*γc^−^*^/*−*^ contribute to the observed effects. Nevertheless we and others have previously shown that NK cells dominate BCR/ABL^+^ tumor surveillance [27,28,29,58,59]. We thus conclude that STAT3 acts as a tumor suppressor in subcutaneously transplanted BCR/ABL^+^ lymphoma in the presence of functional NK cells.

This led us to the following concept: in BCR/ABL^+^ lymphoma STAT3 mediates the recruitment and activation of NK cells. Upon loss of STAT3, NK cells eradicate tumor cells less efficiently resulting in enhanced tumor growth. In BCR/ABL^+^ lymphoma STAT3 functions as a tumor suppressor as it was shown for brain and intestinal tumors [23,24]. In contrast, in other tumor types STAT3 was described to support tumor growth via the down-regulation of pro-inflammatory cytokines. Moreover, blockade of STAT3 enhanced leukocyte infiltration into the tumor and activated innate immune cells such as macrophages and neutrophils resulting in an increased anti-tumor cytotoxicity [60,61]. Taken together, the entity of studies on STAT3 indicates the following picture: STAT3 cannot be generally judged as a tumor suppressor or tumor promoter—the part STAT3 plays depends on the tumor type and the responsible immune effector cell(s). In BCR/ABL^+^ lymphoma, which are under the control of NK cells, STAT3 acts as a tumor suppressor.

## 3. Experimental

### 3.1. Mice

*Mx1-*Cre [62], *Stat3^fl^*^/*fl*^ [38], *Nu*/*Nu* (The Jackson Laboratory, 600 Main Street, Bar Harbor, ME, USA), *Rag2^−^*^/*−*^*γc^−^*^/*−*^ [63] and C57BL/6J wild-type mice were maintained under pathogen-free conditions at the Medical University of Vienna and the University of Veterinary Medicine Vienna. All animal experiments were approved by the institutional ethics committee and conform to Austrian laws (license BMWF-68.205/0218-II/3b/2012).

### 3.2. Generation of BCR/ABL^+^ Cell Lines, *in Vitro* Deletion of Endogenous *Stat3* and shRNA-Mediated Knockdown

To generate BCR/ABL^+^ cell lines, BM cells of the offspring of a *Stat3^fl^*^/*fl*^ × *Stat3^fl^*^/*fl*^ *Mx1*-Cre breeding were transformed retrovirally (by BCR/ABL^p185^ or v-ABL^p160^) and maintained in RPMI-1640 supplemented with 10% FCS, 50 µM β-mercaptoethanol and 100 U/mL penicillin, 100 µg/mL streptomycin (PAA) as previously described [28]. For *Stat3* deletion, stable *Stat3^fl^*^/*fl*^ *Mx1*-Cre BCR/ABL^p185+^ cell lines were incubated for 48 h in 1,000 U/mL recombinant murine interferon-β (IFN-β; PBL Interferon Source, Piscataway, NJ, USA). After two weeks, deletion efficiency was verified by genotyping PCR as described before [8].

For shRNA-mediated knockdown, hairpins targeting *Stat3* were lentivirally transduced into wild-type BCR/ABL^p185+^ and v-ABL^p160+^ cells. The encoded hairpin (expressed in a pLKO.1-puro-vector) anneals with the mRNA region transcribed by exon 11 thereby degrading both splice variants of STAT3 (Stat3α and Stat3β). The TRC clone (TRCN0000071456) was obtained from MISSION^®^ shRNA. A clone containing a validated non-silencing shRNA served as control. Selection of vector-expressing cells was accomplished by puromycin selection (1 µg/mL) for 7 days. Efficiency of shRNA-mediated STAT3 knockdown was verified by western blotting.

### 3.3. Protein Extracts and Western Blotting

Whole cell extracts were performed as previously described [64,65]. For western blotting, proteins (50–100 µg) were separated on a 7% SDS polyacrylamide gel and transferred to nitrocellulose membranes. Membranes were probed with antibodies directed against STAT1 (sc-592, clone M-22), STAT3 (CS#9132), STAT5 (sc-853, clone C-17) and β-actin (A5441, clone AC-15). Immunoreactive bands were visualized by chemiluminescence (20 × LumiGLO^®^ Reagent and 20 × Peroxide, Cell Signaling, , 3 Trask Lane, Danvers, MA, USA).

### 3.4. Transplantation of Tumor Cells

10^5^ BCR/ABL^+^ cells were injected subcutaneously into the flanks of wild-type, *Nu*/*Nu* and *Rag2^−^*^/*−*^*γc^−^*^/*−*^ mice. After 11 days or when tumors reached a maximum size of 3 cm^3^, mice were sacrificed and tumors excised. For flow cytometric analysis tumors were mashed through a 70 µM filter.

### 3.5. Immunohistochemistry

Paraffin-embedded tumor samples were stained with CD31 (Dianova (Warburgstr. 45, Hamburg, Germany), DIA-310, dilution 1:40, 90 min) and Ki67 (Novocastra (Hernalser Hauptstrasse 219, Vienna, Austria), MM1, dilution 1:200, 30 min) according to the manufacturers’ instructions.

### 3.6. Flow Cytometry

Single-cell suspensions were pre-incubated with anti-CD16/CD32 antibodies to prevent non-specific Fc-receptor-mediated binding and then stained with fluorescently labeled antibodies. The following antibodies, all purchased from BD Biosciences, were used: anti-CD3ε (145-2C11), anti-CD49b (DX5), anti-CD4 (L3T4), anti-CD8a (53-6.7), anti-GR1 (RB6-8C5), anti-CD11b (M1/70), anti-B220 (RA3-6B2) and anti-CD19 (1D3).

For cell cycle analysis 10^6^ cells were stained with propidium iodide (50 µg/mL) in a hypotonic lysis solution (0.1% sodium citrate, 0.1% triton X-100, 100 µg/mL RNAse) and incubated at 37 °C for 30 min. Analysis of stained cells was performed using a FACS Canto II flow cytometer equipped with 488, 633 and 405 nm lasers using the FACS Diva software version 6.1.2 (Becton, Dickinson and Company, 1 Becton Drive, Franklin Lakes, NJ, USA).

### 3.7. [^3^H]-Thymidine Incorporation

5 × 10^4^ cells were plated in 96-round-bottom-well plates (in triplicates) in the presence of [^3^H]-thymidine (0.1 μCi/well [0.0037 MBq/well]). After 12 h of incubation, cells were lysed and transferred onto glass fiber filters. Rotiszint^®^ eco plus (ROTH) was added and radioactivity was analyzed in a liquid scintillation counter (Tri-Carb 1900 CA, PerkinElmer, 940 Winter Street, Waltham, MA, USA).

### 3.8. Real-Time PCR Analysis

RNA was isolated using peqGOLD TriFast reagent. First-strand cDNA synthesis was performed using the iSCRIPT cDNA synthesis kit (Bio-Rad Laboratories, 1000 Alfred Nobel Drive, Hercules, CA, USA) according to the manufacturer’s instructions. Real-time PCR was performed on a MyiQTM2 Cycler (BioRad Laboratories) using SsoFastTM EvaGreen^®^Supermix (BioRad Laboratories). The following primers were used (Microsynth Austria, Leberstrasse 20, Vienna, Austria): *Rplp0_F*: 5'-TTCATTGTGGGAGCAGAC-3' and *Rplp0_R:* 5'-CAGCAGTTTCTCCAGAGC-3'; *Il6_F*: 5'-TTCCATCCAGTTGCCTTCTTGG-3' and *Il6_R:* 5'-TTCTCATTTCCACGATTTCCCAG-3'; *Il10_F*: 5'-AGGGTTACTTGGGTTGCCAA-3' and *Il10_R*: 5'-CACAGGGGAGAAATCGATGA-3'; *Ifng_F*: 5'-AAGTGGCATAGATGTGGAAG-3' and *Ifng_R*: 5'-GAATGCATCCTTTTTCGCCT-3'; *Tnfa_F*: 5'-GCGGAGTCCGGGCAGGTCTA-3' and *Tnfa_R*: 5'-GGGGGCTGGCTCTGTGAGGA-3'; *Il17_F*: 5'-CTGCTGAGCCTGGCGGCTAC-3' and *Il17_R*: 5'-CATTGCGGTGGAGAGTCCAGGG-3'; *Ccl5_F*: 5'-CCACTTCTTCTCTGGGTTGG-3' and *Ccl5_R*: 5'-GTGCCCACGTCAAGGAGTAT-3'. Target gene expression was normalized to the house-keeping gene *Rplp0*.

### 3.9. *In Vitro* NK Cell Cytotoxicity Assay

NK cells were isolated from splenocytes via magnetic-activated cell sorting (MACS; anti-DX5 microbeads, Miltenyi Biotec, Friedrich-Ebert-Straße 68, Bergisch Gladbach, Germany) and cultivated for 7 days in RPMI-1640 containing l-glutamine, 10% FCS, 50 µM β-mercaptoethanol, 100 U/mL penicillin, 100 µg/mL streptomycin and 5,000 U/mL recombinant human IL-2 (Proleukin, Novartis International AG, Basel, Switzerland). NK cells were co-cultured with 5 × 10^4^ CFSE-labeled (2.5 mM; CellTrace CFSE Cell Proliferation Kit, Molecular Probes, 3175 Staley Road, Grand Island, NY, USA) target cells at different effector-to-target (E:T) ratios (10:1, 5:1 and 2.5:1) in triplicates. After 24 h, samples were stained with 7-aminoactinomycin D (7-AAD; 0.1 mg; eBioscience, 10255 Science Center Drive, San Diego, CA, USA) for 5 min and analyzed by flow cytometry: % specific lysis = [% 7-AAD^+^CFSE^+^ cells after co-incubation with NK cells] − [% 7-AAD^+^CFSE^+^ cells without addition of NK cells] according to [58].

## 4. Conclusions

Therapeutic agents blocking STAT3 are currently under development and predicted to block tumor progression in a variety of malignancies. We show here that in BCR/ABL^+^ tumors the absence of STAT3 accelerates tumor growth assigning a tumor suppressing function to STAT3 in this disease. We found that STAT3 is required to attract NK cells to the tumor, which restrict and limit tumor growth. In the absence of STAT3 tumor surveillance is significantly impaired. Caution should be taken when targeting STAT3 in lymphoma as it may provoke adverse effects on immune surveillance in these patients.

## References

[1] Hanahan D., Weinberg R.A. (2000). The hallmarks of cancer. Cell.

[2] Weinstein I.B. (2002). Cancer. Addiction to oncogenes—The Achilles heal of cancer. Science.

[3] Luo J., Solimini N.L., Elledge S.J. (2009). Principles of cancer therapy: Oncogene and non-oncogene addiction. Cell.

[4] Bowman T., Garcia R., Turkson J., Jove R. (2000). STATs in oncogenesis. Oncogene.

[5] Yu H., Jove R. (2004). The STATs of cancer—New molecular targets come of age. Nat. Rev. Cancer.

[6] Ling X., Arlinghaus R.B. (2005). Knockdown of STAT3 expression by RNA interference inhibits the induction of breast tumors in immunocompetent mice. Cancer Res..

[7] Bar-Natan M., Nelson E.A., Xiang M., Frank D.A. (2012). STAT signaling in the pathogenesis and treatment of myeloid malignancies. JAK-STAT.

[8] Hoelbl A., Schuster C., Kovacic B., Zhu B., Wickre M., Hoelzl M.A., Fajmann S., Grebien F., Warsch W., Stengl G. (2010). *Stat5* is indispensable for the maintenance of bcr/abl-positive leukaemia. EMBO Mol. Med..

[9] Bromberg J. (2002). Stat proteins and oncogenesis. J. Clin. Investig..

[10] Buettner R., Mora L.B., Jove R. (2002). Activated STAT signaling in human tumors provides novel molecular targets for therapeutic intervention. Clin. Cancer Res..

[11] Azare J., Leslie K., Al-Ahmadie H., Gerald W., Weinreb P.H., Violette S.M., Bromberg J. (2007). Constitutively activated *Stat3* induces tumorigenesis and enhances cell motility of prostate epithelial cells through integrin beta 6. Mol. Cell Biol..

[12] Abdulghani J., Gu L., Dagvadorj A., Lutz J., Leiby B., Bonuccelli G., Lisanti M.P., Zellweger T., Alanen K., Mirtti T. (2008). *Stat3* promotes metastatic progression of prostate cancer. Am. J. Pathol..

[13] Barbieri I., Pensa S., Pannellini T., Quaglino E., Maritano D., Demaria M., Voster A., Turkson J., Cavallo F., Watson C.J. (2010). Constitutively active *Stat3* enhances neu-mediated migration and metastasis in mammary tumors via upregulation of Cten. Cancer Res..

[14] Gough D.J., Corlett A., Schlessinger K., Wegrzyn J., Larner A.C., Levy D.E. (2009). Mitochondrial STAT3 supports Ras-dependent oncogenic transformation. Science.

[15] Zhang Q., Raje V., Yakovlev V.A., Yacoub A., Szczepanek K., Meier J., Derecka M., Chen Q., Hu Y., Sisler J. (2013). Mitochondrial localized *Stat3* promotes breast cancer growth via phosphorylation of serine 727. J. Biol. Chem..

[16] Kim D.J., Angel J.M., Sano S., DiGiovanni J. (2009). Constitutive activation and targeted disruption of signal transducer and activator of transcription 3 (*Stat3*) in mouse epidermis reveal its critical role in UVB-induced skin carcinogenesis. Oncogene.

[17] Weber-Nordt R.M., Egen C., Wehinger J., Ludwig W., Gouilleux-Gruart V., Mertelsmann R., Finke J. (1996). Constitutive activation of STAT proteins in primary lymphoid and myeloid leukemia cells and in Epstein-Barr virus (EBV)-related lymphoma cell lines. Blood.

[18] Lin T.S., Mahajan S., Frank D.A. (2000). STAT signaling in the pathogenesis and treatment of leukemias. Oncogene.

[19] Benekli M., Baer M.R., Baumann H., Wetzler M. (2003). Signal transducer and activator of transcription proteins in leukemias. Blood.

[20] Benekli M., Baumann H., Wetzler M. (2009). Targeting signal transducer and activator of transcription signaling pathway in leukemias. J. Clin. Oncol..

[21] Ecker A., Simma O., Hoelbl A., Kenner L., Beug H., Moriggl R., Sexl V. (2009). The dark and the bright side of *Stat3*: Proto-oncogene and tumor-suppressor. Front. Biosci..

[22] Lee J., Kim J.C.K., Lee S.-E., Quinley C., Kim H., Herdman S., Corr M., Raz E. (2012). Signal transducer and activator of transcription 3 (STAT3) protein suppresses adenoma-to-carcinoma transition in Apcmin/+ mice via regulation of Snail-1 (SNAI) protein stability. J. Biol. Chem..

[23] De la Iglesia N., Konopka G., Puram S.V., Chan J.A., Bachoo R.M., You M.J., Levy D.E., Depinho R.A., Bonni A. (2008). Identification of a PTEN-regulated STAT3 brain tumor suppressor pathway. Genes Dev..

[24] Musteanu M., Blaas L., Mair M., Schlederer M., Bilban M., Tauber S., Esterbauer H., Mueller M., Casanova E., Kenner L. (2010). *Stat3* is a negative regulator of intestinal tumor progression in Apc(Min) mice. Gastroenterology.

[25] Mair M., Zollner G., Schneller D., Musteanu M., Fickert P., Gumhold J., Schuster C., Fuchsbichler A., Bilban M., Tauber S. (2010). Signal transducer and activator of transcription 3 protects from liver injury and fibrosis in a mouse model of sclerosing cholangitis. Gastroenterology.

[26] Vesely M.D., Kershaw M.H., Schreiber R.D., Smyth M.J. (2011). Natural innate and adaptive immunity to cancer. Annu. Rev. Immunol..

[27] Stoiber D., Kovacic B., Schuster C., Schellack C., Karaghiosoff M., Kreibich R., Weisz E., Artwohl M., Kleine O.C., Müller M. (2004). TYK2 is a key regulator of the surveillance of B lymphoid tumors. J. Clin. Investig..

[28] Kovacic B., Stoiber D., Moriggl R., Weisz E., Ott R.G., Kreibich R., Levy D.E., Beug H., Freissmuth M., Sexl V. (2006). STAT1 acts as a tumor promoter for leukemia development. Cancer Cell.

[29] Baron F., Turhan A.G., Giron-Michel J., Azzarone B., Bentires-Alj M., Bours V., Bourhis J.H., Chouaib S., Caignard A. (2002). Leukemic target susceptibility to natural killer cytotoxicity: Relationship with BCR-ABL expression. Blood.

[30] Maghazachi A.A. (2010). Role of chemokines in the biology of natural killer cells. Curr. Top. Microbiol. Immunol..

[31] Kärre K., Ljunggren H.G., Piontek G., Kiessling R., Karre K. (1986). Selective rejection of H-2-deficient lymphoma variants suggests alternative immune defence strategy. Nature.

[32] Raulet D.H., Guerra N. (2009). Oncogenic stress sensed by the immune system: Role of natural killer cell receptors. Nat. Rev. Immunol..

[33] Yu H., Pardoll D., Jove R. (2009). STATs in cancer inflammation and immunity: A leading role for STAT3. Nat. Rev. Cancer.

[34] Lee H., Pal S.K., Reckamp K., Figlin R.A., Yu H. (2011). STAT3: A target to enhance antitumor immune response. Curr. Top. Microbiol. Immunol..

[35] Niu G., Wright K.L., Huang M., Song L., Haura E., Turkson J., Zhang S., Wang T., Sinibaldi D., Coppola D. (2002). Constitutive *Stat3* activity up-regulates VEGF expression and tumor angiogenesis. Oncogene.

[36] Kortylewski M., Yu H. (2008). Role of *Stat3* in suppressing anti-tumor immunity. Curr. Opin. Immunol..

[37] Kortylewski M., Kujawski M., Wang T., Wei S., Zhang S., Pilon-Thomas S., Niu G., Kay H., Mule J., Kerr W.G. (2005). Inhibiting *Stat3* signaling in the hematopoietic system elicits multicomponent antitumor immunity. Nat. Med..

[38] Alonzi T., Maritano D., Gorgoni B., Rizzuto G., Libert C., Poli V. (2001). Essential role of STAT3 in the control of the acute-phase response as revealed by inducible gene inactivation [correction of activation] in the liver. Mol. Cell Biol..

[39] Levy D.E., Lee C. (2002). What does *Stat3* do?. J. Clin. Investig..

[40] Fagard R., Metelev V., Souissi I., Baran-Marszak F. (2013). STAT3 inhibitors for cancer therapy: Have all roads been explored?. JAK-STAT.

[41] Ilaria R.L., van Etten R.A. (1996). P210 and P190(BCR/ABL) induce the tyrosine phosphorylation and DNA binding activity of multiple specific STAT family members. J. Biol. Chem..

[42] Shuai K., Halpern J., ten Hoeve J., Rao X., Sawyers C.L. (1996). Constitutive activation of STAT5 by the BCR-ABL oncogene in chronic myelogenous leukemia. Oncogene.

[43] Steelman L.S., Pohnert S.C., Shelton J.G., Franklin R.A., Bertrand F.E., McCubrey J.A. (2004). JAK/STAT, Raf/MEK/ERK, PI3K/Akt and BCR-ABL in cell cycle progression and leukemogenesis. Leukemia.

[44] Spiekermann K., Pau M., Schwab R., Schmieja K., Franzrahe S., Hiddemann W. (2002). Constitutive activation of STAT3 and STAT5 is induced by leukemic fusion proteins with protein tyrosine kinase activity and is sufficient for transformation of hematopoietic precursor cells. Exp. Hematol..

[45] Aggarwal B.B., Kunnumakkara A.B., Harikumar K.B., Gupta S.R., Tharakan S.T., Koca C., Dey S., Sung B. (2009). Signal transducer and activator of transcription-3, inflammation, and cancer: How intimate is the relationship?. Ann. NY Acad. Sci..

[46] Bromberg J.F., Wrzeszczynska M.H., Devgan G., Zhao Y., Pestell R.G., Albanese C., Darnell J.E. (1999). *Stat3* as an oncogene. Cell.

[47] Levy D.E., Inghirami G. (2006). STAT3: A multifaceted oncogene. Proc. Natl. Acad. Sci. USA.

[48] Ishida Y., Kondo T., Takayasu T., Iwakura Y., Mukaida N. (2004). The essential involvement of cross-talk between IFN-gamma and TGF-beta in the skin wound-healing process. J. Immunol..

[49] Shinozaki M., Okada Y., Kitano A., Ikeda K., Saika S., Shinozaki M. (2009). Impaired cutaneous wound healing with excess granulation tissue formation in TNFalpha-null mice. Arch. Dermatol. Res..

[50] Schall T.J., Bacon K., Toy K.J., Goeddel D.V. (1990). Selective attraction of monocytes and T lymphocytes of the memory phenotype by cytokine RANTES. Nature.

[51] Allavena P., Germano G., Marchesi F., Mantovani A. (2011). Chemokines in cancer related inflammation. Exp. Cell Res..

[52] Taub D.D., Sayers T.J., Carter C.R., Ortaldo J.R. (1995). Alpha and beta chemokines induce NK cell migration and enhance NK-mediated cytolysis. J. Immunol..

[53] Ming W.J., Bersani L., Mantovani A. (1987). Tumor necrosis factor is chemotactic for monocytes and polymorphonuclear leukocytes. J. Immunol..

[54] Vivier E., Raulet D.H., Moretta A., Caligiuri M.A., Zitvogel L., Lanier L.L., Yokoyama W.M., Ugolini S. (2011). Innate or adaptive immunity? The example of natural killer cells. Science.

[55] Orr M.T., Lanier L.L. (2010). Natural killer cell education and tolerance. Cell.

[56] Koch J., Steinle A., Watzl C., Mandelboim O. (2013). Activating natural cytotoxicity receptors of natural killer cells in cancer and infection. Trends Immunol..

[57] Kovanen P.E., Leonard W.J. (2004). Cytokines and immunodeficiency diseases: Critical roles of the gamma(c)-dependent cytokines interleukins 2, 4, 7, 9, 15, and 21, and their signaling pathways. Immunol. Rev..

[58] Putz E.M., Gotthardt D., Hoermann G., Csiszar A., Wirth S., Berger A., Straka E., Rigler D., Wallner B., Jamieson A.M. (2013). CDK8-mediated STAT1-S727 phosphorylation restrains NK cell cytotoxicity and tumor surveillance. Cell Rep..

[59] Zebedin E., Simma O., Schuster C., Putz E.M., Fajmann S., Warsch W., Eckelhart E., Stoiber D., Weisz E., Schmid J.A. (2008). Leukemic challenge unmasks a requirement for PI3Kdelta in NK cell-mediated tumor surveillance. Blood.

[60] Yu H., Kortylewski M., Pardoll D. (2007). Crosstalk between cancer and immune cells: Role of STAT3 in the tumour microenvironment. Nat. Rev. Immunol..

[61] Wang T., Niu G., Kortylewski M., Burdelya L., Shain K., Zhang S., Bhattacharya R., Gabrilovich D., Heller R., Coppola D. (2004). Regulation of the innate and adaptive immune responses by *Stat-3* signaling in tumor cells. Nat. Med..

[62] Kühn R., Schwenk F., Aguet M., Rajewsky K. (1995). Inducible gene targeting in mice. Science.

[63] Cao X., Shores E.W., Hu-Li J., Anver M.R., Kelsall B.L., Russell S.M., Drago J., Noguchi M., Grinberg A., Bloom E.T. (1995). Defective lymphoid development in mice lacking expression of the common cytokine receptor gamma chain. Immunity.

[64] Schreiber E., Matthias P., Müller M.M., Schaffner W. (1989). Rapid detection of octamer binding proteins with “mini-extracts”, prepared from a small number of cells. Nucleic Acids Res..

[65] Hoelbl A., Kovacic B., Kerenyi M.A., Simma O., Warsch W., Cui Y., Beug H., Hennighausen L., Moriggl R., Sexl V. (2006). Clarifying the role of Stat5 in lymphoid development and Abelson-induced transformation. Blood.

